# First-in-human phase 1 dose-escalation results with livmoniplimab, an antibody targeting the GARP:TGF-ß1 complex, as monotherapy and in combination with the anti–PD-1 antibody budigalimab in patients with advanced solid tumors

**DOI:** 10.3389/fonc.2024.1376551

**Published:** 2024-10-29

**Authors:** Toshio Shimizu, John Powderly, Albiruni Abdul Razak, Patricia LoRusso, Kathy D. Miller, Steven Kao, Sarah Kongpachith, Catherine Tribouley, Michelle Graham, Brian Stoll, Maulik Patel, Mohammad Sahtout, Martha Blaney, Rachel Leibman, Talia Golan, Anthony Tolcher

**Affiliations:** ^1^ Department of Experimental Therapeutics, National Cancer Center Hospital, Tokyo, Japan; ^2^ Department of New Experimental Therapeutics and International Cancer New Drug Development Center, Kansai Medical University Hospital, Osaka, Japan; ^3^ Carolina BioOncology Institute, Huntersville, NC, United States; ^4^ Cancer Clinical Research Unit (CCRU), Princess Margaret Cancer Centre, Toronto, ON, Canada; ^5^ Yale Cancer Center, Yale University, New Haven, CT, United States; ^6^ Department of Medicine, Indiana University Melvin and Bren Simon Comprehensive Cancer Center, Indianapolis, IN, United States; ^7^ Department of Medical Oncology, Chris O’Brien Lifehouse, Sydney, NSW, Australia; ^8^ AbbVie Bay Area, South San Francisco, CA, United States; ^9^ Institute of Oncology, Sheba Medical Center, Tel Hashomer, Ramat Gan, Israel; ^10^ Oncology Institute, Sheba Medical Center at Tel-Hashomer, Tel Aviv University, Tel Aviv, Israel; ^11^ New Experimental Therapeutics (NEXT) Oncology, San Antonio, TX, United States

**Keywords:** advanced solid tumors, TGF-ß1, GARP, immunotherapy, anti-PD-1 antibody, combination drug therapy, investigational therapies, tumor microenvironment (TME)

## Abstract

**Background:**

Transforming growth factor (TGF)-ß1 is a pleiotropic cytokine that can promote tumor growth and suppress antitumor immune responses. Latent TGF-ß1 associates with glycoprotein-A repetition predominant (GARP) on the surface of regulatory T cells prior to its activation and release. Livmoniplimab is a monoclonal antibody (mAb) that binds the GARP:TGF-ß1 complex to inhibit activation and release of TGF-ß1. It is in clinical development in combination with budigalimab, an anti-programmed cell death protein 1 Fc-modified mAb. The first-in-human, phase 1, dose-escalation results are presented herein (ClinicalTrials.gov: NCT03821935).

**Methods:**

The dose-escalation phase enrolled adult patients with advanced solid tumors. Patients received escalating doses of livmoniplimab ranging from 3mg to 1500mg, once every 2 weeks (Q2W), as monotherapy or in combination with a 500mg fixed dose of budigalimab Q4W. The primary objective of the dose escalation was to determine the recommended phase 2 dose. Secondary objectives were to assess safety and pharmacokinetics (PK), and exploratory objectives included evaluating preliminary efficacy.

**Results:**

Fifty-seven patients enrolled in the dose escalation: 23 in monotherapy cohorts and 34 in combination therapy cohorts. Dose-limiting toxicities were limited, no maximum tolerated dose was reached, and the maximum administered dose of 1500mg was selected for dose expansion. The most common adverse events reported in monotherapy-treated patients were fatigue, anemia, and nausea, and those in combination therapy-treated patients were pruritus, fatigue, nausea, and anemia. Livmoniplimab exhibited dose-proportional PK, and peripheral blood biomarker data demonstrated saturation of the GARP:TGF-ß1 complex on platelets at livmoniplimab doses within the linear PK range. No objective tumor responses were observed in the monotherapy dose escalation. However, the objective response rate was 15% in the combination dose escalation, with a median response duration of 8.4 months.

**Conclusion:**

Livmoniplimab was well-tolerated as monotherapy and in combination with budigalimab in the dose-escalation phase. Encouraging preliminary efficacy was demonstrated in the combination dose escalation in heavily pretreated patients, supporting further development of this novel drug combination in patients with advanced solid tumors.

## 1 Introduction

Transforming growth factor (TGF)-ß1 is a potent immunomodulatory cytokine that plays a key role in various cellular processes including cell proliferation, epithelial-to-mesenchymal transition and migration, and angiogenesis (1, 2). In oncogenesis, TGF-ß1 signaling pathways are hijacked by cancer cells to promote cancer progression (3, 4). In the tumor microenvironment (TME), TGF-ß1 promotes tumor growth by multiple mechanisms including: suppressing effector T cells, natural killer cells, and dendritic cells; inducing anti-inflammatory macrophage M2 polarization; and promoting tumor fibrosis via induction of cancer-associated fibroblasts, collagen proteins, and other extracellular matrix proteins (5). TGF-ß1 overexpression and signaling in cancer has been associated with poor prognosis and resistance to immune checkpoint inhibitors, including anti-programmed cell death protein 1 (PD-1)/PD-1 ligand 1 (PD-L1) therapies (6, 7).

TGF-ß1 is produced in a latent form, in which mature TGF-ß1 is complexed with a latency-associated peptide, thus preventing the mature TGF-ß1 from binding to its specific receptors and subsequent signaling (8). This latent TGF-ß1 complex associates with various latent TGF-ß binding proteins at the cell surface. One such protein is glycoprotein-A repetition predominant (GARP), expressed on the surface of immune cells, primarily CD4^+^ regulatory T cells (Tregs) and platelets (9, 10), as well as some cancer cells (11–14). GARP binding to latent TGF-ß1 results in localization and concentration of the TGF-ß1 on the surface of immune cells (15), where TGF-ß1 activation and release is regulated by various integrins (15, 16).

Multiple therapeutic strategies have been developed to target TGF-ß expression and signaling, either by broadly targeting all TGF-ß isoforms, specifically targeting TGF-ß1 or TGF-ß2, or targeting the TGF-ß receptor. These include (a) anti-integrin agents that inhibit TGF-ß activation, (b) antibodies or antibody-based biotherapeutics against TGF-ß or its receptors that interfere with ligand-receptor interactions and downstream signaling, (c) small-molecule kinase inhibitors that interfere with TGF-ß receptor kinase activity and signaling, and d) antisense oligonucleotides (17–19). Despite promising preclinical antitumor activity in all cases, these TGF-ß–targeting agents have had mixed success in the clinic: some have failed due to toxicity or insufficient antitumor activity, some demonstrated encouraging preliminary clinical data that have yet to be confirmed in a registrational study, and others remain in early developmental stages. As a result, there is currently no TGF-ß–targeting agent approved in oncology (20). A potential limitation of these therapeutic approaches targeting TGF-ß pathways is that their inhibition of TGF-ß signaling is not specific to the TME. Since TGF-ß1 is a pleiotropic cytokine that is expressed by most cells, systemically blocking TGF-ß1 activity may result in undesirable side effects, and inhibition locally in the TME may be beneficial.

Inhibiting TGF-ß1 activation and release from GARP on the surface of CD4^+^ Tregs is a novel approach to target TGF-ß in a more site-restricted manner. Tregs are immune-suppressing cells that have been associated with poor outcomes in several tumor types (7) and resistance to checkpoint inhibitors (21–23). TGF-ß production by Tregs has been identified as a mechanism of immune suppression within the TME, and GARP may play a key role in facilitating localized TGF-ß release (19). GARP expression and TGF-ß1 release are increased in various solid tumors, including breast cancer (13), lung cancer (13), gastric cancer (12), colon cancer (13), and hepatocellular carcinoma (24).

Antibodies that bind to the GARP:TGF-ß1 complex and inhibit release of active TGF-ß1 were first developed by the laboratory of Prof Sophie Lucas in partnership with Argenx; these antibodies were shown to inhibit Treg immunosuppression in a xenogeneic graft-versus-host disease mouse model (25). Subsequently, the Lucas group demonstrated that antibodies against the mouse GARP:TGF-ß1 complex could overcome resistance to anti–PD-1 agents in a colon carcinoma mouse model and induce T-cell–mediated immunity that protected mice from tumor rechallenge (9).

**Figure 1:**
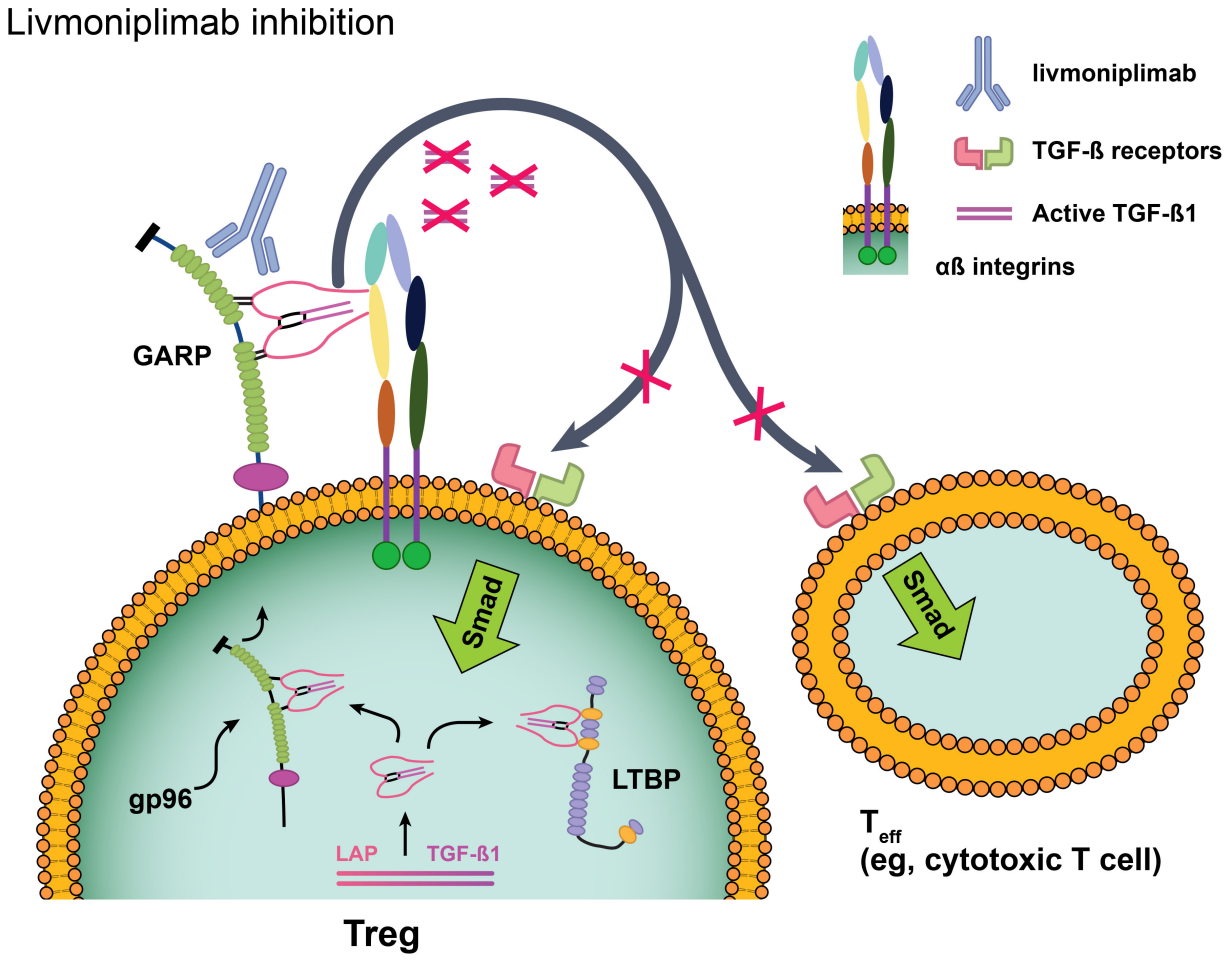
Livmoniplimab targets the GARP:TGF-ß complex and inhibits release of mature, active TGF-ß. Reproduced with edits under the terms of the Creative Commons Attribution 4.0 International License (https://creativecommons.org/licenses/by/4.0/) from Metelli A, Salem M, Wallace CH, Wu BX, Li A, Li X, et al. Immunoregulatory functions and the therapeutic implications of GARP-TGF-ß in inflammation and cancer. *J Hematol Oncol* (2018) 11(1):24 (26). Changes were made to depict the livmoniplimab proposed mechanism of action. GARP, glycoprotein-A repetition predominant; LAP, latency-associated peptide; LTBP, latent TGF-ß binding protein; Smad, mothers against decapentaplegic family of transcription factors; Teff, effector T cell; TGF-ß, transforming growth factor ß; TME, tumor microenvironment; Treg, regulatory T cell.

Livmoniplimab is a first-in-class human immunoglobulin G4/k monoclonal antibody (mAb) that binds to the human GARP:TGF-ß1 complex and inhibits the release of mature TGF-ß1 [**Figure 1** (26)]. It is being developed in combination with budigalimab (also known as ABBV-181), an investigational anti–PD-1 Fc-modified immunoglobulin G1 mAb that has demonstrated safety and efficacy in patients with non-small cell lung cancer and head and neck squamous cell carcinoma (27). Despite the broad application of anti–PD-1 antibodies in solid tumor immunotherapy, a considerable proportion of disease fails to respond to these agents or acquires resistance, and multiple lines of evidence support dual inhibition of PD-1 and TGF-ß as a therapeutic strategy. Mariathasan and colleagues observed that a TGF-ß gene signature in fibroblasts was associated with lack of response to atezolizumab in immune-excluded metastatic urothelial carcinoma (6). When gene expression analyses were performed on multiple cohorts from The Cancer Genome Atlas database, an overlap between markers of T-cell infiltration (typically associated with response to PD-1 blockade) and TGF-ß–related gene signatures was revealed, indicating that TGF-ß may be a mechanism of immune escape in these patients (AbbVie internal data). This concept was corroborated by mouse model data in which increased tumor growth inhibition and reinvigorated CD8^+^ T-cell effector responses were demonstrated in mice treated with a combination of antibodies targeting PD-1 and the GARP:TGF-ß1 complex compared with either antibody alone (9). On the basis of this evidence, a clinical trial was designed to evaluate inhibition of the GARP:TGF-ß1 complex and PD-1. Herein, we present the results from the dose-escalation phase of this first-in-human (FIH) phase 1 study of livmoniplimab as monotherapy and in combination with budigalimab in patients with advanced solid tumors.

## 2 Methods

### 2.1 Study design

This is a phase 1, open-label, FIH, dose-escalation and dose-expansion study. Livmoniplimab was assessed via dose escalation as monotherapy and in combination with budigalimab in patients with locally advanced or metastatic solid tumors. Dose escalation was guided by a Bayesian optimal interval design based on the cumulative number of patients experiencing a dose-limiting toxicity (DLT) at each dose level. Dose expansion was designed to evaluate multiple cohorts of locally advanced or metastatic solid tumors.

For the dose-escalation phase, the primary objective was to determine the recommended phase 2 dose of livmoniplimab monotherapy and in combination with budigalimab. The secondary objective was to assess safety, tolerability, and pharmacokinetics (PK) of livmoniplimab as monotherapy and combined with budigalimab. Exploratory objectives included evaluating the preliminary efficacy of livmoniplimab as monotherapy and in combination with budigalimab and evaluating the pharmacodynamics (PD) and predictive biomarkers associated with PK, safety, and efficacy. The trial was registered with ClinicalTrials.gov (NCT03821935) and was approved by institutional review boards at each participating site prior to initiation. The study was performed in accordance with the International Conference on Harmonization Good Clinical Practice guidelines and the Declaration of Helsinki, with written informed consent obtained from all patients before study enrollment.

The first 2 livmoniplimab monotherapy cohorts, at dose levels 3mg and 10mg, enrolled a single patient. For livmoniplimab monotherapy dose cohorts at 30mg or higher, a minimum of 3 patients were enrolled. The combination dose-escalation phase began after ≥2 monotherapy dose levels were determined to be safe, with a minimum of 3 patients enrolled per cohort. Livmoniplimab was administered via intravenous infusion once every 2 weeks (Q2W), and in combination cohorts, budigalimab was administered at a 500mg fixed dose via intravenous infusion once every 4 weeks (Q4W). Patients in both arms received livmoniplimab, with or without budigalimab, until disease progression or intolerable toxicity.

### 2.2 Study population

The dose-escalation phase required that patients be ≥18 years of age with an advanced solid tumor considered refractory or intolerant to all existing therapies known to provide clinical benefit, unless patients were ineligible for or refused standard therapies. Patients were also required to have Eastern Cooperative Oncology Group performance status of 0 or 1 and adequate bone marrow, renal, hepatic, and coagulation function. Patients with unresolved adverse events (AEs) grade >1 from prior anticancer treatment (except alopecia), with clinically significant uncontrolled conditions or with uncontrolled metastases to the central nervous system, were excluded. Patients were also excluded if they had a history of any of the following: primary immunodeficiency, bone marrow or solid organ transplantation, clinical diagnosis of tuberculosis, active autoimmune disease, inflammatory bowel disease, interstitial lung disease or pneumonitis, myocarditis, Stevens-Johnson syndrome, toxic epidermal necrolysis, or drug reaction with eosinophilia and systemic symptoms.

### 2.3 Safety and efficacy assessments and statistics

Safety endpoints of treatment-emergent AEs (TEAEs; onset on or after the first dose and up to 90 days after the last dose), serious AEs, deaths, and changes in laboratory and vital sign parameters were assessed in all patients who received ≥1 dose of the study drug. DLTs were assessed for a period of 28 days following the first dose of livmoniplimab monotherapy or livmoniplimab and budigalimab combination therapy per the National Cancer Institute Common Terminology Criteria for AEs version 5.0. Patients who did not complete the full 28-day DLT observation period, for any reason other than a DLT, were considered non-DLT evaluable and were replaced at the same dose level. Patients were continuously monitored for known or expected immune-related toxicities.

Efficacy was evaluated per investigator assessments according to Response Evaluation Criteria in Solid Tumors (RECIST) v1.1 every 8 weeks for the first 12 months, then every 12 weeks until disease progression; all patients who received ≥1 dose of the study drug were considered. Patients were allowed to continue treatment beyond progression per RECIST v1.1 if they were absent of symptoms or signs of disease progression and had no decline in Eastern Cooperative Oncology Group performance status. Such patients were then evaluated using the modified RECIST v1.1 criteria for immune-based therapeutics. Objective response rate and its 2-sided 95% Clopper-Pearson (exact) confidence interval were calculated for each cohort on the basis of patients showing complete response or partial response (PR). Median duration of response and its 2-sided 95% confidence intervals were calculated for each cohort.

### 2.4 Platelet GARP:TGF-ß target engagement (TE) assay

Livmoniplimab saturation of the GARP:TGF-ß complex on peripheral blood platelets was assayed by immunostaining of isolated platelet-rich plasma (PRP). Whole blood samples were collected into K2*EDTA vacutainers and shipped to a central laboratory for analysis according to institutional review board-approved ethical guidelines. Blood collection tubes were centrifuged at 150×g for 15 minutes at 4°C without brake. The PRP supernatant layer was carefully collected and aliquoted prior to enumeration by an automated hematology analyzer. The PRP fractions were treated with dimethyl sulfoxide at a final concentration of 6% (v/v) and stored at – 80°C until immunostaining.

For immunostaining, PRP aliquots were thawed briefly at 37°C, washed with assay buffer (1% Human AB serum and 2 mM ethylenediaminetetraacetic acid in 500 mL of phosphate buffer saline), and stained with a platelet-specific mAb conjugated to a fluorescent fluorochrome, CD61-FITC from BioLegend (San Diego, CA). Two additional AbbVie proprietary reagents, 1E7-APC to detect GARP receptor levels and LHG10.6-PE to detect GARP:TGF-ß receptor levels, with accompanying isotype controls were included in the stain mixture to assess target engagement. Mean fluorescence intensities and quantitation beads for APC and PE (Bangs Laboratory) were used to determine GARP and GARP:TGF-ß levels, respectively, on purified platelets. Receptor levels were extrapolated from calibration curves generated from bead mean fluorescence intensity and mean equivalent soluble fluorochrome (MESF) density values. Longitudinal TE values were calculated using the equation: 100 * (1– [LHG10-PE_MESF_ Postdosing – Isotype-PE_MESF_ Postdosing]/[LHG10-PE_MESF_ Baseline – Isotype-PE_MESF_ Baseline]) and plotted using Prism (GraphPad 9). Assay validation and sample processing were conducted by MLM Medical Labs in Memphis, TN, in accordance with AbbVie guidance.

### 2.5 PK and antidrug antibody (ADA) assessments

Serial blood samples for measurements of livmoniplimab and budigalimab concentrations in serum were collected in cycles 1 and 3 prior to infusion, 15 minutes after the end of the respective infusion, and at 2 hours, 4 hours (only for livmoniplimab), 24 hours, 168 hours, and 336 hours, following the end of the respective infusion. PK samples were collected in all other cycles prior to infusion and 15 minutes after the end of the respective infusion. The lower limit of quantitation was 1.63 ng/mL and 50 ng/mL for livmoniplimab and budigalimab, respectively. Livmoniplimab and budigalimab serum concentrations were quantified using a validated bioanalytical assay and analyzed using noncompartmental analysis in Phoenix WinNonlin (version 8.3 Pharsight, Mountain View, CA). Peak serum concentrations, time to peak concentration, area under the curve to 336 hours, and terminal half-life were determined for livmoniplimab and budigalimab. Livmoniplimab and budigalimab blood samples for measurement of ADA were collected predose on day 1 of each cycle with an additional early ADA assessment on day 15 in cycle 1 only. All patients who received ≥1 dose of the study drug and had ≥1 valid postbaseline PK data were included in this analysis.

### 2.6 Exploratory blood PD biomarker assessments

Blood samples for exploratory biomarker assessment by flow cytometry were collected before infusion on day 1, day 8 and 15 of cycle 1, day 1(pre-dose) and 15 of cycle 2, day 1(pre-dose) of cycle 3. Memory T-cell frequencies and Ki67 proliferation were evaluated using validated flow cytometry assays (Covance Inc., USA) on freshly obtained anticoagulated blood as previously described (28).

## 3 Results

### 3.1 Translational PK/PD model to select FIH dose levels of livmoniplimab

A translational PK/PD model was used to predict the human PK of livmoniplimab and the corresponding target occupancy on platelets and tumor-infiltrating lymphocytes (TILs) on the basis of nonclinical data. Briefly, allometric scaling was used to predict the human PK parameters based on those estimated by fitting the data from a single-dose non-Good Laboratory Practice PK/PD study in cynomolgus monkeys to a 2-compartment model with target-mediated saturable clearance. The target engagement parameters estimated on the basis of the model fit were combined with measurements of target levels on platelets and TILs to calculate predicted target occupancy (%GARP-TGFß1 complexes bound by livmoniplimab) in human.

A maximum recommended starting dose for livmoniplimab of 3mg (0.05mg/kg for 60kg body weight) was selected on the basis of the model prediction of ≤80% maximum target occupancy on platelets in the peripheral blood and ≤15% on TILs (assuming the livmoniplimab concentration in the tumor is much less than that in the serum). In addition, the model predicted a duration of target occupancy of >10% on platelets for <5 days postdose at the maximum recommended starting dose. Dose escalations for the next 5 cohorts were based on ~3-fold increases. A maximum dose of 1500mg was selected on the basis of the model prediction of >99% target occupancy on both platelets and TILs. The final livmoniplimab dose levels evaluated were therefore 3mg, 10mg, 30mg, 100mg, 300mg, 1000mg, and 1500mg.

### 3.2 Patient demographics and baseline characteristics

Between March 2019 and February 2022, 23 patients were enrolled in the livmoniplimab monotherapy dose-escalation cohorts (3mg, N=1; 10mg, N=1; 30mg, N=3; 100mg, N=3; 300mg, N=3; 1000mg, N=4; 1500mg, N=8) and 34 patients enrolled in the livmoniplimab and budigalimab dose-escalation cohorts (livmoniplimab 10mg, N=4; 30mg, N=8; 100mg, N=3; 300mg, N=4; 1000mg, N=4; 1500mg, N=11; budigalimab 500mg fixed dose). Patient demographics and baseline disease characteristics are summarized in **Table 1**. Patients with a variety of solid tumors were enrolled in the dose-escalation phase; the most frequent tumor types in the monotherapy cohorts were non-small cell lung cancer (n=4), colorectal (n=3), and ovarian cancer (n=3), and in the combination therapy cohorts were colorectal (n=8), ovarian (n=7), and pancreatic cancer (n=4). Patients in the monotherapy cohorts had received a median of 4 (range 1, 10) prior lines of systemic therapies, and those in the combination therapy cohorts had received a median of 3 (range 0, 10) prior lines of systemic therapies. Eight (35%) and 10 (29%) patients receiving monotherapy and combination therapy, respectively, had received prior anti–PD-1 or anti–PD-L1 therapy.

**Table 1:** Patient demographics and tumor baseline characteristics.

Livmoniplimab Monotherapy (Q2W)								
Livmoniplimab dosage	3mg (N=1)	10mg (N=1)	30mg (N=3)	100mg (N=3)	300mg (N=3)	1000mg (N=4)	1500mg (N=8)	Total (N=23)
**Median age at baseline, years (range)**	78.0 (78, 78)	54.0 (54, 54)	75.0 (47, 77)	71.0 (46, 73)	67.0 (46, 73)	66.5 (36, 74)	65.5 (49, 81)	67.0 (36, 81)
**Sex, n (%)** *Male* *Female*	0 1 (100)	0 1 (100)	0 3 (100)	3 (100) 0	1 (33) 2 (67)	3 (75) 1 (25)	1 (13) 7 (87)	8 (35) 15 (65)
**Race, n (%)** *White* *Black or African American* *Asian*	1 (100) 0 0	1 (100) 0 0	1 (33) 0 2 (67)	3 (100) 0 0	1 (33) 1 (33) 1 (33)	1 (25) 1 (25) 2 (50)	3 (38) 0 5 (63)	11 (48) 2 (9) 10 (43)
**ECOG performance status at baseline, n (%)** 0 1	1 (100) 0	1 (100) 0	2 (67) 1 (33)	1 (33) 2 (67)	1 (33) 2 (67)	1 (25) 3 (75)	4 (50) 4 (50)	11 (48) 12 (52)
**Primary cancer type, n (%)** *TNBC* *Pancreatic* *Urothelial* *HCC* *NSCLC* *Ovarian* *Colorectal* *Breast cancer (non-TNBC)* *Head and neck (HNSCC)* *Other solid tumors^a^*	0 0 0 0 0 1 (100) 0 0 0 0	0 0 0 0 0 1 (100) 0 0 0 0	1 (33) 0 0 0 1 (33) 0 0 0 0 1 (33)	0 0 0 0 1 (33) 0 0 0 0 2 (67)	0 1 (33) 0 0 0 1 (33) 0 0 0 1 (33)	0 0 0 0 0 0 0 0 0 4 (100)	1 (13) 0 0 0 2 (25) 0 3 (38) 1 (13) 0 1 (13)	2 (9) 1 (4) 0 0 4 (17) 3 (13) 3 (13) 1 (4) 0 9 (39)
**Median prior lines of systemic therapy, n (range)**	6.0 (6, 6)	3.0 (3, 3)	5.0 (4, 9)	1.0 (1, 4)	3.0 (3, 10)	2.0 (1, 5)	5.0 (1, 8)	4.0 (1, 10)
**Received prior anti–PD-(L)1 therapy,^b^ n (%)** *Yes* *No*	0 1 (100)	1 (100) 0	1 (33) 2 (67)	1 (33) 2 (67)	0 3 (100)	2 (50) 2 (50)	3 (38) 5 (62)	8 (35) 15 (65)

Livmoniplimab (Q2W) + Budigalimab (500mg Q4W) Combination Therapy							
Livmoniplimab dosage	10mg (N=4)	30mg (N=8)	100mg (N=3)	300mg (N=4)	1000mg (N=4)	1500mg (N=11)	Total (N=34)
**Median age at baseline, years (range)**	50.0 (46, 62)	53.5 (39, 72)	52.0 (50, 63)	63.5 (62, 71)	67.5 (48, 77)	53.0 (20, 70)	57.5 (20, 77)
**Sex, n (%)** *Male* *Female*	2 (50) 2 (50)	6 (75) 2 (25)	1 (33) 2 (67)	1 (25) 3 (75)	0 4 (100)	1 (9) 10 (91)	11 (32) 23 (68)
**Race, n (%)** *White* *Black or African American* *Asian*	3 (75) 1 (25) 0	7 (88) 1 (13) 0	2 (67) 0 1 (33)	3 (75) 0 1 (25)	3 (75) 0 1 (25)	6 (55) 1 (9) 4 (36)	24 (71) 3 (9) 7 (21)
**ECOG performance status at baseline, n (%)** 0 1	4 (100) 0	2 (25) 6 (75)	3 (100) 0	3 (75) 1 (25)	0 4 (100)	8 (73) 3 (27)	20 (59) 14 (41)
**Primary cancer type, n (%)** *TNBC* *Pancreatic* *Urothelial* *HCC* *NSCLC* *Ovarian* *Colorectal* *Breast cancer (non-TNBC)* *Head and neck (HNSCC)* *Other solid tumors^a^*	0 1 (25) 0 0 0 1 (25) 2 (50) 0 0 0	0 1 (13) 0 0 0 0 2 (25) 1 (13) 0 4 (50)	0 0 0 0 0 1 (33) 2 (67) 0 0 0	0 1 (25) 0 0 0 0 2 (50) 0 0 1 (25)	0 0 0 0 1 (25) 0 0 1 (25) 0 2 (50)	0 1 (9) 1 (9) 0 0 5 (46) 0 0 0 4 (36)	0 4 (12) 1 (3) 0 1 (3) 7 (21) 8 (24) 2 (6) 0 11 (32)
**Median prior lines of systemic therapy, n (range)**	3.0 (1, 7)	4.0 (2, 10)	6.0 (2, 10)	2.5 (2, 3)	3.5 (2, 6)	3.0 (0, 7)	3.0 (0, 10)
**Received prior anti–PD-(L)1 therapy,^b^ n (%)** *Yes* *No*	1 (25) 3 (75)	3 (38) 5 (62)	1 (33) 2 (67)	2 (50) 2 (50)	1 (25) 3 (75)	2 (18) 9 (82)	10 (29) 24 (71)

^a^Other solid tumor types include sarcoma, mesothelioma, endometrial cancer, uterine cancer, gastric/gastroesophageal junction adenocarcinoma, prostate cancer, papillary adenocarcinoma, gastrointestinal stromal tumor, hemangiopericytoma, renal cell carcinoma, adrenocortical carcinoma, orbital sebaceous gland cancer, and ampullary adenocarcinoma. ^b^Patient received either prior anti–PD-1 or anti–PD-L1 therapy.

ECOG, Eastern Cooperative Oncology Group; HCC, hepatocellular carcinoma; HNSCC, head and neck squamous cell carcinoma; NSCLC, non-small cell lung cancer; PD-1, programmed cell death protein 1; PD-L1, PD-1 ligand 1; Q2W, once every 2 weeks; Q4W, once every 4 weeks; TNBC, triple-negative breast cancer.

### 3.3 Drug exposure

In the livmoniplimab monotherapy dose escalation, patients received a median of 2 cycles and the median duration of exposure was 43 days. Patients in the combination therapy dose escalation received a median of 2.5 cycles, and were exposed to livmoniplimab and budigalimab for a median duration of 54 days and 44 days, respectively. At the time of data cutoff on 30 March 2023, all 23 patients enrolled in the monotherapy dose-escalation cohorts discontinued treatment, most commonly due to disease progression (87%); 33 (97%) patients enrolled in the combination therapy dose escalation discontinued treatment, with disease progression being the most common reason (59%). Details of patient drug exposure are summarized in **Supplementary Table S1**.

### 3.4 PK and ADA analysis

**Figure 2:**
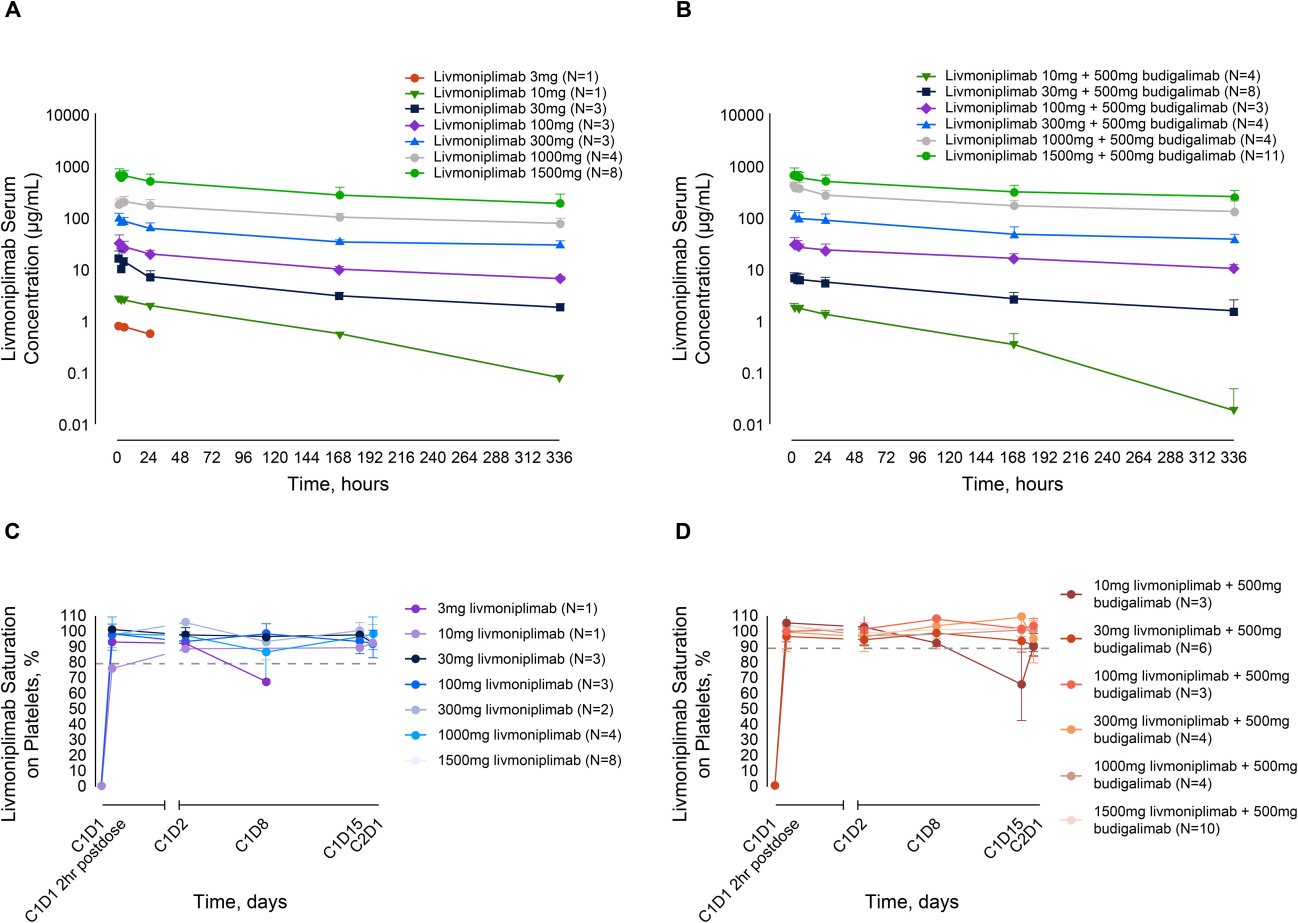
Livmoniplimab PK and TE profiles. **(A)**: Livmoniplimab Q2W PK profile for monotherapy dose escalation cohorts. **(B)**: Livmoniplimab Q2W PK profile for livmoniplimab and budigalimab combination therapy cohorts. **(C)**: GARP:TGF-β platelet TE for livmoniplimab Q2W monotherapy dose escalation cohorts. **(D)**: GARP:TGF-β platelet TE for livmoniplimab Q2W and budigalimab combination therapy cohorts. On the basis of the assay validation characterization (data not shown), complete TE was established at 80% saturation. C, cycle; D, day; GARP, glycoprotein-A repetition predominant; PK, pharmacokinetics; Q2W, once every 2 weeks; TE, target engagement; TGF-ß, transforming growth factor ß.

As of August 2022, all 57 patients enrolled in the dose-escalation phase who received livmoniplimab monotherapy or combination therapy with budigalimab have preliminary PK data available. Mean livmoniplimab serum concentration-versus-time profiles from cycle 1 (after the first dose) from monotherapy and combination therapy escalation cohorts are presented in **Figures 2A** and **B**, respectively. The preliminary mean PK parameters for the monotherapy cohorts are presented in **Supplementary Table S2A** and for the combination cohorts in **Supplementary Table S2B**. Livmoniplimab exhibits dose-proportional PK across the dose range of 30 to 1500mg, where complete GARP:TGF-ß1 target saturation in circulation over the treatment period was observed (**Figures 2C, D**). Approximately 2-fold accumulation was observed on a Q2W administration schedule in cycle 3 compared with cycle 1. Livmoniplimab or budigalimab PK (data on file) was not impacted by their coadministration. No treatment-emergent ADAs were reported for either livmoniplimab (N=32) at doses >30mg Q2W or budigalimab (N=34).

### 3.5 GARP:TGF-ß platelet TE

Since activated Tregs that upregulate the GARP:TGF-ß1 complex are challenging to detect in circulation, a surrogate TE assay was developed and validated on purified peripheral blood platelets to determine the extent of livmoniplimab saturation of the GARP:TGF-ß1 complex after intravenous administration. In **Figures 2C** and **D**, longitudinal plots depict the degree of saturation as early as 2 hours postdosing with livmoniplimab in the indicated dosing cohorts receiving monotherapy and combination therapy, respectively. The single patient who received 3mg livmoniplimab monotherapy attained complete saturation at 2hr postdosing, which then minimally desaturated at cycle 1 day 8. In contrast, all higher monotherapy dosing cohorts sustained complete saturation in circulation after livmoniplimab administration across the 2-week dosing interval. In the combination therapy arm, all dosing cohorts attained complete saturation 2hr postdosing with livmoniplimab; only the lowest combination cohort receiving 10mg livmoniplimab recorded partial desaturation at cycle 1 day 15.

### 3.6 PD biomarkers

**Figure 3:**
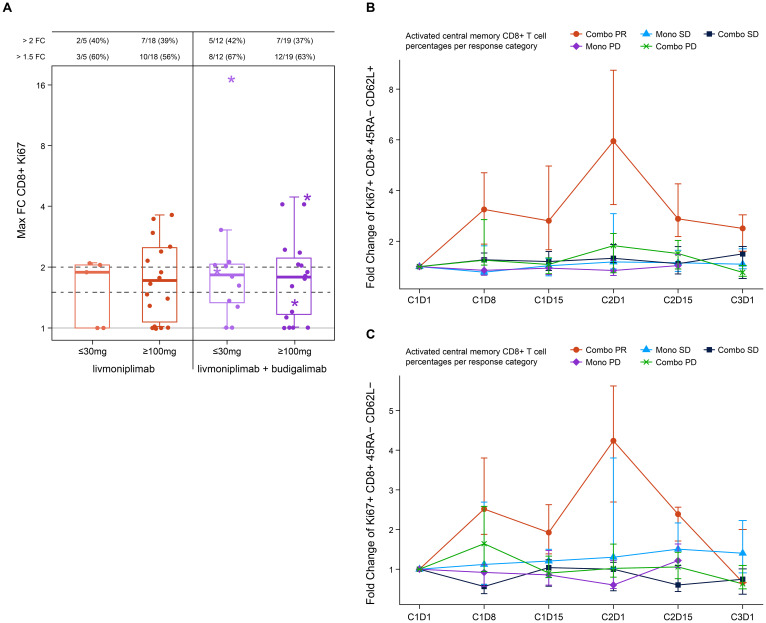
Pharmacodynamic changes induced by livmoniplimab measured by immunophenotyping. **(A)** Activated Ki67^+^ CD8^+^ T cells frequency fold change in monotherapy and combination therapy arms. Star denotes clinical responders. **(B)** Fold change of Ki67^+^ CD8^+^ 45RA^-^ CD62L^+^ activated central memory T-cell frequencies. **(C)** Fold change of Ki67^+^ CD8^+^ 45RA^-^ CD62L^-^ activated central memory T-cell frequencies. Combo, combination therapy; FC, fold change; Mono, monotherapy; PD, progressive disease; PR, partial response; SD, stable disease.

Blood PD biomarkers were longitudinally evaluated by flow cytometry and analyzed according to dose and clinical response status. An increase in proliferating Ki67^+^ CD8^+^ T cells post-treatment was noted in both monotherapy and combination therapy arms with some of the largest increases associated with clinical responders in the combination arms at both low and higher doses (**Figure 3A**). Further analysis of the clinical responders from the combination arms revealed an increase in activated central memory and central effector T cells post-treatment with a peak at C2D1 when compared with patients who had stable disease or patients who experienced progression upon treatment in either monotherapy or combination therapy arms (**Figures 3B, C**). Soluble markers, including cytokines and TGF-ß1, were measured in circulation, and modest changes were observed that were independent of the dose or response (data not shown).

### 3.7 Safety

Safety results from dose escalation are summarized in **Table 2**. In total, 22 (96%) patients receiving livmoniplimab monotherapy experienced a TEAE; the most common TEAEs were fatigue (44%), anemia (35%), and nausea (30%). Livmoniplimab treatment-related AEs (TRAEs) were observed in 16 (70%) patients, with fatigue (22%) and anemia (13%) the most common. Grade 3 or 4 TEAEs occurred in 9 (39%) patients, with the most common being anemia (9%) and atrial fibrillation (9%). One monotherapy-treated patient reported a serious AE deemed related to the study drug – dermatitis, after receiving 1500mg livmoniplimab. Two (9%) patients had a TRAE resulting in treatment interruption, including thrombocytopenia and dermatitis each in 1 patient; no patients had a TRAE resulting in discontinuation in the monotherapy dose escalation.

**Table 2:** Safety.

Livmoniplimab Monotherapy (Q2W)								
Livmoniplimab dosage	3mg (N=1)	10mg (N=1)	30mg (N=3)	100mg (N=3)	300mg (N=3)	1000mg (N=4)	1500mg (N=8)	Total (N=23)
**Any TEAE, n (%)^a,b^** *Fatigue* *Anemia* *Nausea* *Tumor pain* *Diarrhea* *Increased AST* *Decreased appetite* *Vomiting* *Increased GGT* *Dizziness* *Disease progression* *Arthralgia* *Peripheral edema* *Dehydration* *Increased blood alkaline phosphatase* *Pain* **TRAEs leading to either study drug interruption** **TRAEs leading to either study drug discontinuation**	1 (100) 0 0 1 (100) 1 (100) 0 0 1 (100) 1 (100) 0 0 0 1 (100) 0 0 0 0 0 0	1 (100) 1 (100) 0 0 0 0 0 0 1 (100) 0 1 (100) 0 0 1 (100) 0 0 0 0 0	3 (100) 1 (33) 2 (67) 1 (33) 0 0 1 (33) 0 0 1 (33) 1 (33) 1 (33) 0 0 0 0 0 0 0	3 (100) 3 (100) 0 2 (67) 1 (33) 2 (67) 0 1 (33) 1 (33) 0 2 (67) 0 1 (33) 0 0 0 1 (33) 0 0	3 (100) 1 (33) 1 (33) 1 (33) 0 1 (33) 0 0 0 0 0 0 0 0 1 (33) 0 1 (33) 1 (33) 0	3 (75) 0 1 (25) 0 2 (50) 1 (25) 1 (25) 0 0 1 (25) 0 0 0 1 (25) 1 (25) 1 (25) 1 (25) 0 0	8 (100) 4 (50) 4 (50) 2 (25) 2 (25) 1 (13) 2 (25) 2 (25) 1 (13) 2 (25) 0 2 (25) 1 (13) 1 (13) 1 (13) 2 (25) 0 1 (13) 0	22 (96) 10 (44) 8 (35) 7 (30) 6 (26) 5 (22) 4 (17) 4 (17) 4 (17) 4 (17) 4 (17) 3 (13) 3 (13) 3 (13) 3 (13) 3 (13) 3 (13) 2 (9) 0
**Any grade 3 or 4 TEAE, n (%)^c^** *Anemia* *Atrial fibrillation*	0 0 0	0 0 0	1 (33) 1 (33) 1 (33)	0 0 0	1 (33) 1 (33) 0	2 (50) 0 0	5 (63) 0 1 (13)	9 (39) 2 (9) 2 (9)
**Any SAEs related to study drugs, n (%)** *Dermatitis*	0 0	0 0	0 0	0 0	0 0	0 0	1 (13) 1 (13)	1 (4) 1 (4)

Livmoniplimab (Q2W) + Budigalimab (500mg Q4W) Combination Therapy							
Livmoniplimab dosage	10mg (N=4)	30mg (N=8)	100mg (N=3)	300mg (N=4)	1000mg (N=4)	1500mg (N=11)	Total (N=34)
**Any TEAE, n (%)^a,b^** *Pruritis* *Fatigue* *Nausea* *Anemia* *Maculopapular rash* *Diarrhea* *Malignant neoplasm progression* *Rash* *Increased AST* *Arthralgia* *Dry skin* *Increased ALT* *Constipation* *Decreased appetite* *Headache* *Dehydration* *Vomiting* *Pyrexia* *Peripheral edema* *Tumor pain* *Cough* *Hypomagnesemia* *Increased blood creatinine* *Myalgia* *Ascites* *Abdominal pain* *Dyspnea* **TRAEs leading to either study drug interruption** **TRAEs leading to either study drug discontinuation**	4 (100) 3 (75) 0 0 1 (25) 1 (25) 0 0 0 0 0 2 (50) 0 0 0 0 1 (25) 0 0 0 0 1 (25) 0 1 (25) 0 0 0 0 0 0	8 (100) 2 (25) 5 (63) 4 (50) 3 (38) 3 (38) 1 (13) 3 (38) 1 (13) 2 (25) 1 (13) 1 (13) 2 (25) 2 (25) 1 (13) 0 2 (25) 2 (25) 3 (38) 2 (25) 2 (25) 0 1 (13) 2 (25) 1 (13) 1 (13) 1 (13) 2 (25) 1 (13) 1 (13)	3 (100) 0 2 (67) 2 (67) 0 0 1 (33) 0 1 (33) 1 (33) 1 (33) 0 1 (33) 0 1 (33) 1 (33) 0 0 0 0 1 (33) 1 (33) 1 (33) 0 1 (33) 0 0 1 (33) 1 (33) 0	4 (100) 2 (50) 3 (75) 1 (25) 1 (25) 0 3 (75) 2 (50) 2 (50) 0 0 0 0 1 (25) 0 2 (50) 1 (25) 2 (50) 1 (25) 0 1 (25) 1 (25) 0 0 0 1 (25) 0 0 0 0	4 (100) 2 (50) 1 (25) 3 (75) 3 (75) 1 (25) 0 1 (25) 0 0 1 (25) 0 0 0 1 (25) 0 0 0 0 1 (25) 0 1 (25) 0 0 1 (25) 1 (25) 0 0 1 (25) 0	11 (100) 7 (64) 3 (27) 4 (36) 5 (46) 4 (36) 3 (27) 1 (9) 3 (27) 4 (36) 4 (36) 4 (36) 3 (27) 3 (27) 3 (27) 3 (27) 2 (18) 1 (9) 1 (9) 2 (18) 1 (9) 1 (9) 3 (27) 1 (9) 1 (9) 1 (9) 0 1 (9) 6 (55) 4 (37)	34 (100) 16 (47) 14 (41) 14 (41) 13 (38) 9 (27) 8 (24) 7 (21) 7 (21) 7 (21) 7 (21) 7 (21) 6 (18) 6 (18) 6 (18) 6 (18) 6 (18) 5 (15) 5 (15) 5 (15) 5 (15) 5 (15) 5 (15) 4 (12) 4 (12) 4 (12) 1 (3) 4 (12) 9 (27) 5 (15)
**Any grade 3 or 4 TEAE, n (%)^c^** *Anemia* *Malignant neoplasm progression* *Decreased neutrophil count* *Increased ALT* *Increased AST* *Diarrhea* *Tumor pain*	3 (75) 0 0 1 (25) 0 0 0 0	4 (50) 0 2 (25) 0 1 (13) 1 (13) 0 1 (13)	1 (33) 0 0 0 0 0 0 0	4 (100) 0 1 (25) 0 0 0 2 (50) 0	3 (75) 2 (50) 0 1 (25) 0 0 0 0	8 (73) 2 (18) 0 1 (9) 1 (9) 1 (9) 0 1 (9)	23 (68) 4 (12) 3 (9) 3 (9) 2 (6) 2 (6) 2 (6) 2 (6)
**Any SAEs related to study drugs, n (%)** *Increased ALT* *Diabetic ketoacidosis* *Arthralgia* *Autoimmune enteropathy* *Decreased ejection fraction* *Enterocolitis* *Hypotension* *Malaise*	0 0 0 0 0 0 0 0 0	1 (13) 0 0 0 1 (13) 0 0 0 0	0 0 0 0 0 0 0 0 0	0 0 0 0 0 0 0 0 0	0 0 0 0 0 0 0 0 0	4 (36) 1 (9) 1 (9) 1 (9) 0 1 (9) 1 (9) 1 (9) 1 (9)	5 (15) 1 (3) 1 (3) 1 (3) 1 (3) 1 (3) 1 (3) 1 (3) 1 (3)

^a^Occurring in >10% of total patients. ^b^TEAE defined as AEs with onset on or after the first dose and up to 90 days after the last dose date. ^c^Occurring in >5% of total patients. Preferred terms were coded using MedDRA dictionary version 26.0. A patient who reports 1 or more events under the same preferred term is counted only once in that preferred term.

AE, adverse event; ALT, alanine aminotransferase; AST, aspartate aminotransferase; GGT, gamma-glutamyl transferase; MedDRA, Medical Dictionary for Regulatory Activities; Q2W, once every 2 weeks; Q4W, once every 4 weeks; SAE, serious adverse event; TEAE, treatment-emergent adverse event; TRAE, treatment-related adverse event.

In the combination therapy dose escalation, 34 (100%) patients reported TEAEs, with the most common being pruritus (47%), fatigue (41%), nausea (41%), and anemia (38%). Livmoniplimab TRAEs were reported in 25 (74%) patients, the most common being pruritus (35%), maculopapular rash (27%), and fatigue (24%). Budigalimab TRAEs were reported in 24 (71%) patients, with pruritus (35%), maculopapular rash (27%), and fatigue (24%) the most common. Grade 3 or 4 TEAEs were reported in 23 (68%) patients; anemia (12%), malignant neoplasm progression, and decreased neutrophil count (9% each) were the most common. Study drug-related serious AEs were experienced by 5 (15%) patients receiving combination therapy, with no single term reported in more than 1 patient. Nine (27%) patients had an AE related to either livmoniplimab or budigalimab resulting in treatment interruption, with the most common being maculopapular rash, in 4 patients. Five (15%) patients had a TRAE resulting in discontinuation in the combination therapy dose escalation, including maculopapular rash in 2 patients and pruritus, urticaria, and nephritis each in 1 patient.

No patients experienced a DLT in the livmoniplimab monotherapy dose escalation; 1 patient (3%) in the combination dose escalation experienced a DLT of increased alanine aminotransferase. There were no deaths related to either livmoniplimab or budigalimab. The maximum tolerated dose for livmoniplimab as monotherapy or in combination with budigalimab was not reached, and the maximum administered dose of 1500mg was selected for dose expansion.

### 3.8 Efficacy

**Figure 4:**
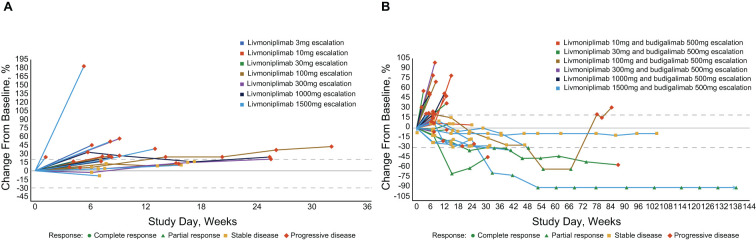
Percentage change in target lesion sum diameter measurements from baseline over time per investigator assessment in response-evaluable set (efficacy-evaluable patients defined as patients who have received at least 1 dose of study drug and have either had at least 1 postdose tumor assessment or discontinued treatment due to AE, progressive disease, or death); per RECIST v1.1 and iRECIST. **(A)** Livmoniplimab monotherapy (Q2W) cohorts (N=22). **(B)** Livmoniplimab (Q2W) and budigalimab combination therapy cohorts (N=34). → Denotes patients still on treatment. One patient did not have on-study tumor measurement data due to early death. AE, adverse event; iRECIST, modified RECIST v1.1 criteria for immune-based therapeutics; Q2W, once every 2 weeks; RECIST, Response Evaluation Criteria in Solid Tumors.

Antitumor efficacy per investigator assessment is shown in **Table 3**. In the monotherapy cohorts, no objective responses were observed; 7 (30%) and 14 (61%) patients experienced stable disease and progressive disease, respectively. In the combination therapy cohorts, the confirmed objective response rate was 15%; 5 (15%), 9 (27%), and 18 (53%) patients had PR, stable disease, and progressive disease, respectively. Tumor response to study drug(s), measured as percentage change from baseline target lesions over time per assessment by the investigator, is depicted for each response-evaluable patient in dose escalation in **Figure 4**. The median duration of objective response for patients treated with combination therapy was 8.4 months.

**Table 3:** Confirmed response per RECIST v1.1 as assessed by investigator.

Livmoniplimab Monotherapy (Q2W)								
Livmoniplimab dosage	3mg (N=1)	10mg (N=1)	30mg (N=3)	100mg (N=3)	300mg (N=3)	1000mg (N=4)	1500mg (N=8)	Total (N=23)
**Objective response rate (CR + PR)^a^** *N (%)* *95% CI^b^*	0 (-, -)	0 (-, -)	0 (-, -)	0 (-, -)	0 (-, -)	0 (-, -)	0 (-, -)	0 (-, -)
**Best overall response per RECIST v1.1^c^** *CR* *PR* *SD^d^* *PD* *Not evaluable* *Not assessed^e^*	0 0 0 1 (100) 0 0	0 0 0 1 (100) 0 0	0 0 0 3 (100) 0 0	0 0 2 (67) 1 (33) 0 0	0 0 2 (67) 1 (33) 0 0	0 0 0 3 (75) 0 1 (25)	0 0 3 (38) 4 (50) 0 1 (13)	0 0 7 (30) 14 (61) 0 2 (9)
**Duration of objective response^f^** *Median (months)* *95% CI^g^*	NR (-, -)	NR (-, -)	NR (-, -)	NR (-, -)	NR (-, -)	NR (-, -)	NR (-, -)	NR (-, -)

Livmoniplimab (Q2W) + Budigalimab (500mg Q4W) Combination Therapy								
Livmoniplimab dosage	10mg (N=4)	30mg (N=8)	100mg (N=3)	300mg (N=4)	1000mg (N=4)	1500mg (N=11)	Total (N=34)
**Objective response rate (CR + PR)^a^** *N (%)* *95% CI^b^*	0 (-, -)	2 (25) (3.2, 65.1)	1 (33) (0.8, 90.6)	0 (-, -)	0 (-, -)	2 (18) (2.3, 51.8)	5 (15) (5.0, 31.1)
**Best overall response per RECIST v1.1^c^** *CR* *PR* *SD^d^* *PD* *Not evaluable* *Not assessed^e^*	0 0 3 (75) 1 (25) 0 0	0 2 (25) 2 (25) 4 (50) 0 0	0 1 (33) 0 2 (67) 0 0	0 0 0 3 (75) 0 1 (25)	0 0 0 4 (100) 0 0	0 2 (18) 4 (36) 4 (36) 1 (9) 0	0 5 (15) 9 (27) 18 (53) 1 (3) 1 (3)
**Duration of objective response^f^** *Median (months)* *95% CI^g^*	NR (-, -)	7.5 (3.65, -)	5.5 (-, -)	NR (-, -)	NR (-, -)	NR (-, -)	8.4 (3.65, -)

^a^Confirmed objective response based on 2 consecutive response assessments at least 28 days apart. ^b^From exact binomial distribution. ^c^Based on response assessment visits prior to subsequent anticancer therapy. ^d^Based on response assessment visit at least 35 days after first dose of study drug. ^e^Patients discontinued study with no postbaseline response assessment (or scan), or patients recently enrolled and did not reach the first postbaseline tumor assessment time point yet and captured under “Other reason for not assessed” category. ^f^Duration of response is defined as the time from the date of first documented CR or PR to the documented date of PD or death, whichever occurs first. Patients who neither progressed nor died or received subsequent anticancer therapy are censored at the last evaluable disease assessment. Patients with subsequent anticancer therapy are censored at the date of last tumor assessment prior to the start of the new therapy. ^g^Based on Kaplan-Meier estimates.

CI, confidence interval; CR, complete response; NR, not reached; PD, progressive disease; PR, partial response; Q2W, once every 2 weeks; Q4W, once every 4 weeks; RECIST, Response Evaluation Criteria in Solid Tumors version 1.1; SD, stable disease.

Responses were observed in multiple solid tumor types across several livmoniplimab dose levels, ranging from 30mg to 1500mg, in combination with a 500mg fixed dose of budigalimab. One responder was a patient with PD-1–naive gastroesophageal junction adenocarcinoma enrolled in the 30mg livmoniplimab combination cohort. Two responders had colorectal cancer; one was microsatellite stable, PD-1 naive, and was enrolled in the 30mg livmoniplimab combination cohort, and another, who was microsatellite instability low (retrospective tumor tissue testing by whole exome sequencing at AbbVie), was enrolled in the 100mg livmoniplimab combination cohort and received, and responded to, prior PD-1 and cytotoxic T-lymphocyte antigen 4 combination checkpoint inhibitor therapy. The last 2 responders were patients with PD-1–naive ovarian cancer treated with 1500mg livmoniplimab combination therapy.

An additional 5 patients who were enrolled in combination therapy dose-escalation cohorts had durable stable disease for approximately 6 months or longer; one of these patients received 10mg livmoniplimab and 4 received 1500mg. These include 1 patient with PD-1–relapsed microsatellite-stable colorectal cancer who had experienced stable disease previously with a combination of anti–PD-1 and anti-cytotoxic T-lymphocyte antigen 4, 1 patient with PD-1–naive alveolar sarcoma, 1 patient with PD-1–relapsed urothelial cancer who had stable disease to prior anti–PD-1, and 2 patients with PD-1–naive ovarian cancer. One of the patients with ovarian cancer converted to an unconfirmed PR after almost a year on study before discontinuing due to an AE. Interestingly, these patients with ovarian cancer who had durable stable disease or PRs had granulosa cell histology, a tumor type for which the importance of TGF-ß signaling in tumorigenesis has been previously demonstrated (29), thus warranting further investigation.

## 4 Discussion

Many novel therapeutics targeting different components of the TGF-ß signaling pathway have entered the clinic to date and remain in various stages of clinical development. Galunisertib and vactosertib are small-molecule TGF-ß receptor 1 kinase inhibitors that have been evaluated in several solid tumor types as monotherapy and in combination with anti–PD-1 or anti–PD-L1 antibodies, radiation therapy, or chemotherapy. Galunisertib development appears to have been discontinued following limited-efficacy data readouts (30–32). While some clinical responses have been observed with vactosertib, the contribution of components has not been published to date (33–36). LY3022859, a small-molecule inhibitor that targets the TGF-ß receptor 2, was discontinued following uncontrolled cytokine release (37).

Different mAbs targeting the TGF-ß pathway have been tested in the clinic as well. NIS793 is a TGF-ß inhibitory mAb being developed in combination with spartalizumab, an anti–PD-1 mAb, or chemotherapy. Clinical responses to NIS793 were observed in a phase 1 dose-escalation and dose-expansion study (38). NIS793 continues to be investigated in a phase 3 study in combination with chemotherapy in metastatic pancreatic ductal adenocarcinoma (39) and in a phase 2 study in colorectal cancer (40). Fresolimumab is another anti–TGF-ß mAb that demonstrated limited response during early clinical trials in patients with solid tumors, including renal cell carcinoma and melanoma (41). However, development of this mAb appears to have been discontinued in oncology. Another TGF-ß–targeting mAb, SAR439459, was discontinued after a FIH study due to lack of efficacy and a substantial risk of bleeding, particularly in patients with hepatocellular carcinoma (42). Utilizing a different approach instead of targeting mature TGF-ß, SRK-181 is a mAb specific for the latent form of TGF-ß1, to prevent its activation. It was well-tolerated and showed preliminary efficacy in a phase 1 trial in advanced solid tumors (43).

Therapeutic modalities beyond small molecules and antibodies have been employed as well. Bintrafusp alfa is a bifunctional fusion protein comprising a human anti–PD-L1 antibody fused to the soluble extracellular domain of TGF-ß receptor II and referred to as a “TGF-ß trap.” This novel therapeutic generated much excitement when preliminary data demonstrated a high objective response rate in PD-L1–high non-small cell lung cancer and other solid tumors (44, 45). Unfortunately, these early results failed to replicate in later phase 2 and phase 3 studies (46–49). Some trials of bintrafusp alfa have yet to report results, including several National Cancer Institute-sponsored and single-institution studies, according to ClinicalTrials.gov. Cilengitide is an αvß3 and αvß5 integrin inhibitor evaluated in solid tumors, including glioblastoma and head and neck squamous cell carcinoma, that failed to improve overall survival in randomized phase 2 and 3 studies (50, 51). Antisense oligonucleotides have been developed to block TGF-ß1 or TGF-ß2, with the latter entering the clinic for solid tumors including glioma (17, 18).

Livmoniplimab is a first-in-class antibody that targets the GARP:TGF-ß1 complex to block the release of active TGF-ß1. It has a differentiated mechanism compared with other antibodies, small molecules, and protein- or nucleotide-based therapeutics targeting TGF-ß that have entered the clinic to date. Livmoniplimab specifically inhibits TGF-ß1 in a GARP-dependent context, which may increase the therapeutic index and/or the tumor-selectivity of this antibody compared with agents that target all TGF-ß isoforms or broadly target TGF-ß1. Whereas bintrafusp alfa requires co-localization of TGF-ß and PD-1 ligands and targets these proteins with a fixed 1:1 dose ratio, the combination of livmoniplimab and budigalimab allows for independent inhibition of the immunosuppressive TGF-ß1 and PD-1 pathways and dose optimization of each agent for sustained target engagement. In addition, targeting TGF-ß1 derived from GARP-expressing Tregs may focus livmoniplimab drug activity on tumors in which Tregs are elevated.

This FIH dose-escalation study of livmoniplimab as monotherapy and in combination with budigalimab enrolled patients with advanced solid tumors who had received a median of 3–4 prior lines of therapy. Peripheral blood biomarker data demonstrated that saturation of the GARP:TGF-ß1 complex on circulating platelets occurred at livmoniplimab doses within the linear PK range (30mg–1500mg), with no treatment-emergent ADA reported for either livmoniplimab (at 30mg to 1500mg range) or budigalimab. The modest changes in TGF-ß1 levels in circulation observed following treatment with livmoniplimab may be difficult to interpret due to the different sources and forms of TGF-ß and may require development of more specific assays. Overall, the clinical PK/PD data observed in the dose escalation aligned with the values predicted in preclinical modeling. Livmoniplimab demonstrated a tolerable safety profile as monotherapy and in combination with budigalimab. The most common TEAEs in monotherapy-treated patients were fatigue, anemia, and nausea, and those in combination therapy-treated patients were pruritus, fatigue, nausea, and anemia. DLTs were limited and no maximum tolerated dose was reached. The maximum administered dose of livmoniplimab was selected for the dose-expansion phase, in order to generate additional biomarker, safety, and efficacy data in cohorts of prespecified solid tumors and select doses for future dose optimization studies.

Encouraging preliminary efficacy was observed in the combination dose escalation in heavily pretreated, advanced solid tumors. While no radiographic responses were observed with livmoniplimab monotherapy, this is consistent with preclinical data in mouse models with GARP:TGF-ß1 surrogate antibodies demonstrating little to no monotherapy activity (de Streel et al., 2020 (9) and AbbVie internal data). Of 34 patients enrolled in the combination dose escalation, 5 patients (15%) experienced confirmed objective responses per investigator RECIST v1.1 assessment, with a median duration of response of 8.4 months. Responses and durable stable disease were observed across multiple solid tumor types including gastroesophageal junction adenocarcinoma, colorectal adenocarcinoma, ovarian cancer, alveolar sarcoma, and urothelial cancer in both PD-1–naive patients and those with prior anti–PD-1 exposure. Livmoniplimab was assessed in solid tumor models preclinically and in patients with advanced solid tumors in this phase 1 study. However, TGF-ß1 dysregulation has been described for hematologic cancers and there are reports of elevated Tregs associated with poor prognosis in these cancers (52–54). Thus, it remains possible that livmoniplimab would exhibit clinical activity beyond solid tumors, particularly in hematologic cancers with elevated GARP-expressing Tregs.

Overall, these dose-escalation data demonstrate encouraging clinical activity and tolerable safety with livmoniplimab, a novel GARP:TGF-ß1 mAb, in combination with budigalimab, an anti–PD-1 Fc-modified mAb. Clinical activity was observed across a range of livmoniplimab doses, from 30mg to 1500mg, where linear PK and target saturation of platelets in circulation was observed. However, it is anticipated that livmoniplimab doses higher than 30mg would be required to achieve sufficient exposure in the TME for complete TE of GARP:TGF-ß1 complex on relevant cell types and to inhibit local, active TGF-ß1 release in a sustained manner across solid tumor indications. Further exploration of this novel drug combination is warranted, with the dose-expansion phase and dose-optimization studies currently enrolling patients with multiple solid tumor types.

## Data Availability

AbbVie is committed to responsible data sharing regarding the clinical trials we sponsor. This includes access to anonymized, individual, and trial-level data (analysis data sets), as well as other information (eg, protocols, clinical study reports, or analysis plans), as long as the trials are not part of an ongoing or planned regulatory submission. This includes requests for clinical trial data for unlicensed products and indications. These clinical trial data can be requested by any qualified researchers who engage in rigorous, independent, scientific research, and will be provided following review and approval of a research proposal, Statistical Analysis Plan, and execution of a Data Sharing Agreement. Data requests can be submitted at any time after approval in the US and Europe and after acceptance of this manuscript for publication. The data will be accessible for 12 months, with possible extensions considered. For more information on the process or to submit a request, visit the following link: https://vivli.org/ourmember/abbvie/.

## Glossary

%CV: coefficient of variation
ADA: antidrug antibody
AE: adverse event
ALT: alanine aminotransferase
AR: accumulation ratio
AST: aspartate aminotransferase
C: cycle
CI: confidence interval
CR: complete response
D: day
DLT: dose-limiting toxicity
ECOG: Eastern Cooperative Oncology Group
FIH: first-in-human
GARP: glycoprotein-A repetition predominant
GGT: gamma-glutamyltransferase
HCC: hepatocellular carcinoma
HNSCC: head and neck squamous cell carcinoma
iRECIST: modified RECIST v1.1 criteria for immune-based therapeutics
LAP: latency-associated peptide
LTBP: latent TGF-β binding protein
mAb: monoclonal antibody
MedDRA: Medical Dictionary for Regulatory Activities
MESF: mean equivalent soluble fluorochrome
NR: not reached
NSCLC: non-small cell lung cancer
PD: pharmacodynamics
PD-1: programmed cell death protein 1
PD-L1: PD-1 ligand 1
PK: pharmacokinetics
PR: partial response
PRP: platelet-rich plasma
Q2W: once every 2 weeks
Q4W: once every 4 weeks
RECIST: Response Evaluation Criteria in Solid Tumors
SAE: serious adverse event
SD: standard deviation
Smad: mothers against decapentaplegic family of transcription factors
t_1/2_: terminal phase elimination half-life
TE: target engagement
TEAE: treatment-emergent adverse event
T_eff_: effector T cell
TGF: transforming growth factor
TIL: tumor-infiltrating lymphocyte
TME: tumor microenvironment
TNBC: triple-negative breast cancer
TRAE: treatment-related adverse event
Treg: regulatory T cell
