# Corrigendum: First-in-human phase 1 dose-escalation results with livmoniplimab, an antibody targeting the GARP: TGF-ß1 complex, as monotherapy and in combination with the anti–PD-1 antibody budigalimab in patients with advanced solid tumors

**DOI:** 10.3389/fonc.2024.1544394

**Published:** 2025-01-31

**Authors:** Toshio Shimizu, John Powderly, Albiruni Abdul Razak, Patricia LoRusso, Kathy D. Miller, Steven Kao, Sarah Kongpachith, Catherine Tribouley, Michelle Graham, Brian Stoll, Maulik Patel, Mohammad Sahtout, Martha Blaney, Rachel Leibman, Talia Golan, Anthony Tolcher

**Affiliations:** ^1^ Department of Experimental Therapeutics, National Cancer Center Hospital, Tokyo, Japan; ^2^ Department of New Experimental Therapeutics and International Cancer New Drug Development Center, Kansai Medical University Hospital, Osaka, Japan; ^3^ Carolina BioOncology Institute, Huntersville, NC, United States; ^4^ Cancer Clinical Research Unit (CCRU), Princess Margaret Cancer Centre, Toronto, ON, Canada; ^5^ Yale Cancer Center, Yale University, New Haven, CT, United States; ^6^ Department of Medicine, Indiana University Melvin and Bren Simon Comprehensive Cancer Center, Indianapolis, IN, United States; ^7^ Department of Medical Oncology, Chris O’Brien Lifehouse, Sydney, NSW, Australia; ^8^ AbbVie Bay Area, South San Francisco, CA, United States; ^9^ Institute of Oncology, Sheba Medical Center, Tel Hashomer, Ramat Gan, Israel; ^10^ Oncology Institute, Sheba Medical Center at Tel-Hashomer, Tel Aviv University, Tel Aviv, Israel; ^11^ New Experimental Therapeutics (NEXT) Oncology, San Antonio, TX, United States

**Keywords:** advanced solid tumors, TGF-ß1, GARP, immunotherapy, anti-PD-1 antibody, combination drug therapy, investigational therapies, tumor microenvironment (TME)

In the published article, there were errors in **Figure 4** as published. The graph included incorrect labeling of livmoniplimab doses for a few patients, including for the patient with deepest response (corrected from livmoniplimab 100mg to livmoniplimab 1500mg) in **Figure 4B**. The corrected **Figure 4** and its caption appear below.

**Figure 4:**
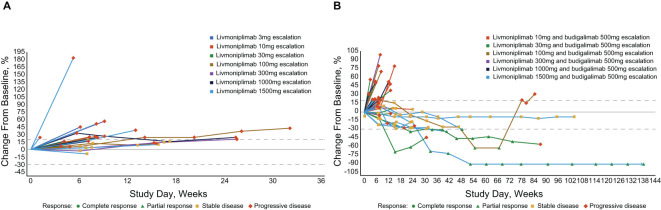
Percentage change in target lesion sum diameter measurements from baseline over time per investigator assessment in response-evaluable set (efficacy-evaluable patients defined as patients who have received at least 1 dose of study drug and have either had at least 1 postdose tumor assessment or discontinued treatment due to AE, progressive disease, or death); per RECIST v1.1 and iRECIST. **(A)** Livmoniplimab monotherapy (Q2W) cohorts (N=22). **(B)** Livmoniplimab (Q2W) and budigalimab combination therapy cohorts (N=34). → Denotes patients still on treatment. One patient did not have on-study tumor measurement data due to early death. AE, adverse event; iRECIST, modified RECIST v1.1 criteria for immune-based therapeutics; Q2W, once every 2 weeks; RECIST, Response Evaluation Criteria in Solid Tumors.

The authors apologize for this error and state that this does not change the scientific conclusions of the article in any way. The original article has been updated.

