# Supplementary material for: First-in-human phase 1 dose-escalation results with livmoniplimab, an antibody targeting the GARP:TGF-ß1 complex, as monotherapy and in combination with the anti–PD-1 antibody budigalimab in patients with advanced solid tumors
Source: Front Oncol. 2024 Oct 29;14:1376551. doi: 10.3389/fonc.2024.1376551 (PMC11555770; doi:10.3389/fonc.2024.1376551)
Supplement: Supplementary file 1 [file Table1.docx]

Supplementary Material

# Supplementary Tables

| **Table S1. Drug Exposure** | | | | | | | | | | |
| --- | --- | --- | --- | --- | --- | --- | --- | --- | --- | --- |
| **Livmoniplimab Monotherapy (Q2W)** | | | | | | | | | | |
| **Livmoniplimab dosage** |  | **3mg  (N=1)** | **10mg**  **(N=1)** | | **30mg**  **(N=3)** | **100mg**  **(N=3)** | **300mg (N=3)** | **1000mg**  **(N=4)** | **1500mg**  **(N=8)** | **Total**  **(N=23)** |
| **Median duration of livmoniplimab, days (range)** |  | 29  (29, 29) | 43  (43, 43) | | 15  (15, 15) | 101  (43, 225) | 99  (64, 151) | 45  (1, 178) | 29  (1, 111) | 43  (1, 225) |
| **Median number of livmoniplimab cycles, n (range)** |  | 2 (2, 2) | 2 (2, 2) | | 1 (1, 1) | 4 (2, 8) | 4 (2, 5) | 2 (1, 5) | 2 (1, 5) | 2 (1, 8) |
| **Livmoniplimab treatment discontinuation, n (%)**  *Adverse event*  *Lost to follow-up*  *Physician decision*  *Progressive disease*  *Withdrew consent*  *COVID-19 infection*  *Other* |  | 1 (100)  0  0  0  1 (100)  0  0  0 | 1 (100)  0  0  0  1 (100)  0  0  0 | | 3 (100)  0  0  0  3 (100)  0  0  0 | 3 (100)  0  0  0  3 (100)  0  0  0 | 3 (100)  0  0  0  3 (100)  0  0  0 | 4 (100)  1 (25)  0  0  3 (75)  0  0  0 | 8 (100)  0  0  1 (13)  6 (75)  1 (13)  0  0 | 23 (100)  1 (4)  0  1 (4)  20 (87)  1 (4)  0  0 |
| **Livmoniplimab (Q2W) + Budigalimab (500mg Q4W) Combination Therapy** | | | | | | | | | | |
| **Livmoniplimab dosage** |  | **10mg**  **(N=4)** | | **30mg**  **(N=8)** | | **100mg**  **(N=3)** | **300mg (N=4)** | **1000mg**  **(N=4)** | **1500mg**  **(N=11)** | **Total**  **(N=34)** |
| **Median duration of livmoniplimab, days (range)** |  | 93  (30, 191) | | 64  (1, 625) | | 51  (43, 639) | 36  (1, 45) | 29  (1, 78) | 169  (1, 946) | 54  (1, 946) |
| **Median duration of budigalimab, days (range)** |  | 86  (30, 191) | | 57  (1, 625) | | 29  (29, 639) | 29  (1, 30) | 15  (1, 64) | 155  (1, 918) | 44  (1, 918) |
| **Median number of cycles of either study drug, n (range)** |  | 4 (2, 7) | | 3 (1, 19) | | 2 (2, 22) | 2 (1, 2) | 1.5 (1, 3) | 6 (1, 27) | 2.5 (1, 27) |
| **Discontinuation of either study drug, n (%)**  *Adverse event*  *Lost to follow-up*  *Physician decision*  *Progressive disease*  *Withdrew consent*  *COVID-19 infection*  *Other* |  | 4 (100)  1 (25)  0  1 (25)  2 (50)  0  0  0 | | 8 (100)  1 (13)  0  0  5 (63)  2 (25)  0  0 | | 3 (100)  0  0  0  3 (100)  0  0  0 | 4 (100)  0  0  1 (25)  3 (75)  0  0  0 | 4 (100)  1 (25)  0  0  3 (75)  0  0  0 | 10 (91)  2 (18)  0  0  4 (36)  2 (18)  0  2 (18) | 33 (97)  5 (15)  0  2 (6)  20 (59)  4 (12)  0  2 (6) |
| Q2W, once every 2 weeks; Q4W, once every 4 weeks. | | | | | | | | | | |

| **Table S2A. Preliminary Geometric Mean (Mean, %CV) Pharmacokinetic Parameters of Livmoniplimab (Escalation Monotherapy)** | | | | | | | | | | | | | |
| --- | --- | --- | --- | --- | --- | --- | --- | --- | --- | --- | --- | --- | --- |
| **Parameter (Unit)** | **Dose (mg)** | | | | | | | | | | | | |
|  | | **3** | | **10** | | **30** | | **100** | | **300** | | **1000** | **1500** |
| **Cycle 1** | | | | | | | | | | | | | |
| N | 1 | | 1 | | 3 | | 3 | | 3 | | 4 | | 8 |
| T_max_^a^ (h) | 1.3 | | 1.3 | | 1.3 (1.3-5.0) | | 1.3 (1.3-3.0) | | 3.0 (1.3-3.0) | | 5.0 (5.0-25.0) | | 1.3 (1.3-5.0) |
| C_max_ (µg/mL) | 0.802 | | 2.79 | | 16.8 (17.4, 33) | | 31.8 (33.7, 41) | | 93.6 (95.2, 23) | | 204 (208, 22) | | 691 (714, 28) |
| AUC_tau_ (µg•day/mL) | 2.39 | | 12.3 | | 61.3 (64.2, 38) | | 173 (174, 10) | | 578 (585, 18) | | 1560 (1590, 26) | | 4270 (4560, 39) |
| t_1/2_^b^ (day) | 1.9 | | 2.8 | | 5.37 ± 1.5 | | 8.21 ± 1.5 | | 10.8 ± 2.1 | | 19.6 ± 10.6 | | 8.93 ± 1.9 |
|  | |  | |  | |  | |  | |  | |  |  |
| **Cycle 3** | | | | | | | | | | | | | |
| N | -- | | -- | | -- | | 2 | | 2 | | 1 | | 2 |
| C_max_ (µg/mL) | -- | | -- | | -- | | 37.0, 49.6 | | 172, 152 | | 556 | | 1580, 1540 |
| AUC_tau_ (µg•day/mL) | -- | | -- | | -- | | 340, 405 | | 1570, 1400 | | 4400 | | 12200, 16800 |
| AR C_max_^c^ | -- | | -- | | -- | | 1.17, 2.35 | | 1.81, 1.3 | | 2.26 | | 2.00, 1.84 |
| AR AUC_tau_^d^ | -- | | -- | | -- | | 1.75, 2.48 | | 2.29, 2.35 | | 2.99 | | 2.09, 2.38 |
| ^a^Median (min-max); calculated from start of infusion.  ^b^Harmonic mean ± pseudo SD.  ^c^AR C_max_ is calculated from individual values reported for cycle 1 and cycle 3 for individual patients.  ^d^AR AUC_tau_ is calculated from individual values reported for cycle 1 and cycle 3 for individual patients.  N = 2 or 1 presented as individual value(s).  %CV, coefficient of variation; AR, mean accumulation ratio; AUC_tau_, area under the serum concentration-time curve for every-2-week (Q2W) dosing (eg, 336hr); C_max_, maximum observed serum concentration; SD, standard deviation; t_1/2_, terminal phase elimination half-life; T_max_, time to maximum serum concentration. | | | | | | | | | | | | | |

| **Table S2B. Preliminary Geometric Mean (Mean, %CV) Pharmacokinetic Parameters of Livmoniplimab (Escalation Combination With Budigalimab Cohorts)** | | | | | | |
| --- | --- | --- | --- | --- | --- | --- |
| **Parameter (Unit)** | **Dose (mg)** | | | | | |
|  | **10** | **30** | **100** | **300** | **1000** | **1500** |
| **Cycle 1** | | | | | | |
| N | 4 | 8 | 3 | 4 | 4 | 11 |
| T_max_^a^ (h) | 2.1 (1.3-5.0) | 1.3 (1.3-25.0) | 1.3 (1.3-3.0) | 1.3 (1.3-3.0) | 1.3 (1.3-5.0) | 1.3 (1.3-5.0) |
| C_max_ (µg/mL) | 1.87 (1.88, 14) | 7.01 (7.13, 20) | 32.3 (32.6, 17) | 105 (108, 26) | 397 (403, 21) | 667 (697, 32) |
| AUC_tau_ (µg•day/mL) | 7.22 (7.56, 34) | 44.3 (46.0, 32)^d^ | 237 (241, 21) | 722 (754, 33) | 2520 (2600, 30) | 4680 (4870, 29) |
| t_1/2_^b^ (day) | 2.38 ± 0.43^c^ | 5.77 ± 3.6^d^ | 10.2 ± 1.0 | 8.35 ± 4.0 | 7.55 ± 3.6 | 9.25 ± 5.7 |
| **Cycle 3** | | | | | | |
| N | 3 | 4 | 1 | -- | 1 | 6 |
| C_max_ (µg/mL) | 3.32 (3.66, 52) | 12.6 (13.4, 40) | 97.2 | -- | 622 | 1260 (1290, 23) |
| AUC_tau_ (µg•day/mL) | 10.9 (13.4, 61) | 102 (110, 49) | 417 | -- | 5870 | 12600 (12900, 24) |
| AR C_max_ | 1.67 (1.88, 58) | 1.66 (1.69, 26) | 3.59^e^ | -- | 1.74^e^ | 1.76 (1.84, 32) |
| AR AUC_tau_ | 1.33 (1.47, 47) | 2.08 (2.10, 16) | 2.29^f^ | -- | 2.24^f^ | 2.47 (2.50, 17) |
| ^a^Median (min-max); calculated from start of infusion.  ^b^Harmonic mean ± pseudo SD.  ^c^N=3.  ^d^N=7.  ^e^AR C_max_ is calculated from individual value reported for cycle 1 and cycle 3 for individual patient.  ^f^AR AUC_tau_ is calculated from individual value reported for cycle 1 and cycle 3 for individual patient.  N = 2 or 1 presented as individual value(s).  %CV, coefficient of variation; AR, mean accumulation ratio; AUC_tau_, area under the serum concentration-time curve for every-2-week (Q2W) dosing (eg, 336hr); C_max_, maximum observed serum concentration; SD, standard deviation; t_1/2_, terminal phase elimination half-life; T_max_, time to maximum serum concentration. | | | | | | |
